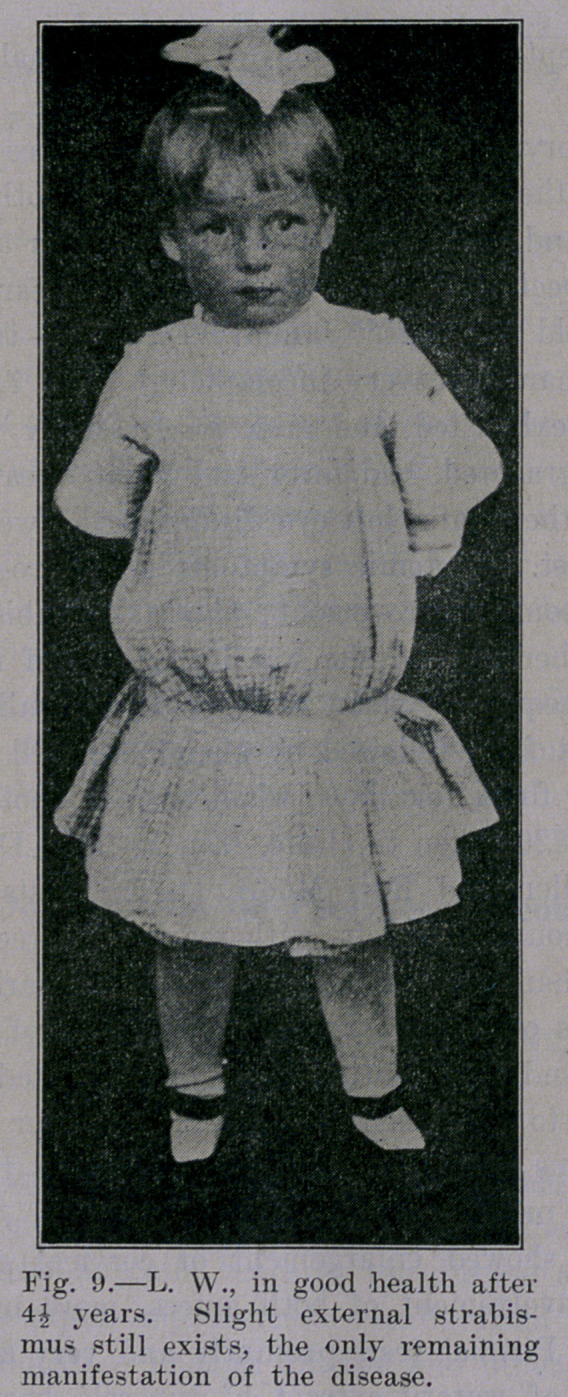# Report of Four Cases of What Appeared to Be Tuberculous Meningitis with Apparent Permanent Arrestment

**Published:** 1914-12

**Authors:** Chas. C. Browning

**Affiliations:** Professor of Diseases of the Chest, University of Southern California, Los Angeles


### TEXAS MEDICAL JOURNAL

Dr. F. E. Daniel, Founder. Established July, 1885

MRS. F. E. DANIEL, - Publisher and Managing Editor

Published Monthly.—Subscription, $1.00 a Year.

VOL. XXX AUSTIN, DECEMBER, 1914 NO. 6

The publisher is not responsible for views of contributors

### Original Articles.

### Report of Four Cases of What Appeared to be Tuberculous Meningitis with Apparent Permanent Arrestment.

BY CHAS. C. BROWNING, M. D., PROFESSOR OF DISEASES OF THE CHEST, UNIVERSITY OF SOUTHERN CALIFORNIA, LOS ANGELES.

Tuberculous meningitis is probably always secondary to active process in other tissues. The meningeal involvement may be general or localized, acute, subacute, or chronic. The gravity of the meningeal infection may be such that the primary focus may be quite overlooked or cease to be of importance from a prognostic standpoint.

It may accompany an attack of acute general miliary tuberculosis, in which the general condition is so grave that the final outcome may not be materially influenced by the meningitis, but the existence of meningeal involvement is always sufficient cause for grave prognosis. Sufficient numbers of cases, however, have been reported of prolonged arrestment of activity, or cures, to show that, while such termination is not frequent, it is possible.

I have collected seventy-six such cases, most of which have been reported within ten years.

These are sufficient to encourage me to report the following cases:

Case I.—In July, 1885 (twenty-nine years ago), I was called to see A. R., near Carthage, Ill., age 4 years, whose parents had died of tuberculosis before she was one year of age, both having an active tuberculous condition before she was born, she having been cared for in a small house with them until their death. The patient had had, and still had at the time of the present illness, enlarged cervical lymphatic glands. The signs and symptoms were those of acute meningitis. The case ran the ordinary course of this disease for about four weeks, during which time three physicians saw her in consultation with me. All agreed that it was probably a case of tubercular meningitis. At the end of the fourth week the symptoms began gradually to subside, a tedious convalescence terminating in apparent recovery. After about a year the patient passed from my observation and was not seen

Fig. 1.—L. W., photograph taken July 18, 1909, when the child was 2 years 7 months old. Note evidence of emaciation.

again until two years ago when she moved to California from Illinois and came to my office. Her health was apparently good, she having had no return of former symptoms. At last account she was teaching school in one of the counties in the central part of the State.

I am quite aware that this case lacks the scientific data which would be required at this time for diagnosis of tuberculous meningitis, and were it not that other cases have been reported of which there could be no doubt of the nature of the illness, I should regard the diagnosis in this particular case as probably incorrect. I report it at this time, leaving persons interested to draw their own conclusions.

Case II.—H. G., age 18, domestic, entered the County Hospital (via ambulance) May 12, 1912.

Family History.—One paternal and one maternal grandparent died of tuberculosis, otherwise history was negative.

Past History.—Patient gave a history of poor health all her life.

Fig. 2.—L. W., photograph taken 20 days later than Fig. 1. Note continuance of emaciation. Apparent slight flatness left side of face.

She had had all the children's diseases in severe form, also typhoid fever and pleurisy, the latter in 1911, six months previously. Menstrual periods began at 13, had always been scanty, irregular, and extremely painful. There was an indefinite history of "heart trouble" in 1911.

Present Illness.—Began four days before entrance to hospital when patient states she "became cold and stiff." For some time previous there had been some loss of weight, fever, cough and expectoration (muco-purulent), night sweats and shortness of breath. Patient complained of severe abdominal pains, loss of appetite and obstinate constipation.

Physical Examination.—Of the chest revealed evidence of active pulmonary tuberculosis in both upper lobes. Temperature irregular, 97-101, frequently from 98-99.5; pulse 70-100. Heart sounds weak and irregular, mitral regurgitant murmur.

Fig. 3.—Photograph showing the condition of patient L. W., Sept. 26, 48 days after first ocular symptoms were manifest. There had been improvement of general physical condition but steady progress of focal manifestations.

Vaginal examination gave extreme tenderness and mass on right side.

Laboratory Examination.—Urine was negative. Sputum showed slight mixed infection. No tubercle bacilli. Cutaneous test actively positive.

Operation on June 7, 1912, twenty-six days after entering hospital, which consisted of an appendectomy, plastic work on both ovaries, and a curettage.

The clinical-pathological report was as follows: Sclerotic condition of ovaries and endometritis—evidence of tuberculosis in appendix and ovaries.

About the middle of July patient began to complain of headache, which soon became persistent and gradually increased in

Fig. 4.—L. W., photograph one month later than Fig. 3. Note improved general condition. Less spasm of muscles of neck and back. Still pronounced ptosis, flatness of left side of face, limp condition of right hand and foot.

severity, with some remissions and exacerbations. The temperature range was generally low, frequently 97-98, occasionally taking a sharp rise to 100, 101, or over, for a short time. The pulse was erratic, varying from 70 to 150. Is reported to have had some rigidity of muscles of neck for short intervals early in August. On August 12th had severe opisthotonos, convulsive seizures, loss of consciousness and retention of urine. Pulse ran to 150, temperature 99. Noguchi test for butyric acid in spinal fluid positive. Pulse dropped to 100 and temperature to 98 following withdrawal of fluid. About ten days later symptoms again became active. Removal of fluid failed to give relief. Succinamide of mercury, gr. 1, given August 28. Apparently slight relief from symptoms. Repeated with 1.2 grains August 31. This was followed by marked

Fig. 5.—L. W., fifteen days later, improvement continues. Ptosis much improved, improvement less marked in left eye. Note storm on right side of face and calm on left.

relief of symptoms. With slight show of return of symptoms this was repeated September 7, gr. 1, and 16, gr. 8. Patient left hospital September 17, 1912. At last report was without marked return of symptoms.

This was apparently a case which was subacute from the beginning, the first symptoms having become manifest about six weeks following operation on tuberculous organs.

Case III.—E. M. W. Referred from the office of Dr. H. G.

Brainerd by Dr. Stephen Smith. The following is abstract of history from Dr. Brainerd's office: December 30, 1910. E. M. W. Female. Single. Age 32. Bookkeeper. Normal weight 140 pounds. Has lost weight rapidly of late, most during the past three weeks.

Complains of weakness, numbness of left hand, and "nervousness."

History.—Always delicate. Severe malaria in childhood. Suf-

Fig. 6.—L. W., eleven days later. Continued improvement. Note healthy appearance of brothers—the other two children in the family.

fered severely with facial neuralgia for several years. Lupus of face for twelve years—three foci. Treated with X-ray and Finsen light. Remains well after nine years. Typhoid fever, 1908. Pulmonary hemorrhage, 1909, one year ago. Weight reduced to 122 pounds. Improved, some gain in weight. slight cough, no fever until about three weeks ago, when began to lose weight rapidly.

About three weeks ago, December 10, stooped over, and was unable to arise without help. Soon felt better. Next morning sister (who is nurse) found her lying with arms extended and rigid, fingers clenched around thumbs, was very restless and nervous. Four days later loss of consciousness accompanied by mumbling and heavy breathing, no twitching. After two weeks right arm relaxed, but the hand is numb and weak with some pain along back of hand. Left arm less improved, much tenderness inner side of left arm; numbness and weakness much more pronounced in left hand than right.

Mental condition impaired since present exacerbation began

Fig. 7.—L. W., showing condition Jan. 6, 1910, eighteen days following onset of measles.

three weeks ago. During this time has had chills at irregular intervals and fever reaching as high as 101 degrees. Appetite abnormally great, bowels regular, sleeps poorly.

Present Condition.—December 30, 1910, much demented. Tests of sensation not wholly reliable on account of mental confusion, but all seem to be more blunted in left arm and hand than right, station fair, co-ordination very poor. Same is true in less degree of left leg and foot. Wrist and elbow jerk greater on left side than right, also knee and ankle jerk greater on left side, but less than in upper limb.

According to notes, the above conditions appeared to increase slightly with remissions and exacerbations until January 15, 1911, when patient was taken with hiccough. Right eyelid dropped. Left eye turned in. Conscious. Muttering speech. About fifteen minutes following onset of these symptoms patient fell asleep for a time followed by increased mental dullness, with increased impairment of left hand, arm, and lower limb—intensity being in the order named. Slight amelioration of symptoms after twenty-four hours.

Similar attacks occurred on the 19th and 31st inst., the last one being preceded by metallic taste in mouth.

January 25 Wassermann test by Dr. Warden reporter negative. February 13 paresthesia around mouth, talks with difficulty, tongue thick. Drinks with difficulty. February 20 very restless, distressed and depressed. Growing weaker. Weakness more pronounced on left side, most left hand. Paresthesia around mouth more troublesome. Talks thickly. Sleeps with mouth oypen. Temperature variable, but subnormal most of the time, 97-98. Pulse 90-100. Believed to be tuberculous.

November 11, 1911, about three months after beginning of symptoms of present attack, she presented herself at my office, accompanied by sister. She was much depressed mentally; would sob and tears start when I attempted to obtain history from her, and appeared to be incapable of answering questions intelligently. Complained of numbness in hands, arms, and lower limbs, most pronounced on left side—would rub left hand and say "it has gone away." There was some ptosis. Her facial expression was blank and stupid; would wander away if not watched. Muscle power was much reduced in both hands, almost absent in left; gait unsteady, imperfect use of both lower limbs, the use of left limb being much more impaired than right. Would fall easily, if not assisted while walking. Reflexes as given above. Weight 137 pounds; loss of sixteen pounds during three months. Temperature range, oral, 97-98. Pulse 80-90. Physical examination of chest showed evidence of fibrosis in the upper lobes of both lungs, most extensive in right where cavity signs were apparent about second and third ribs. Cutaneous tests promptly positive, lumbar puncture not made.

Watery extract of tubercle bacilli was used, beginning March 6 with .001 mg. doses, which were gradually increased to .01 mg. in about two months. Focal reactions were easily excited, as evidenced by temporary exacerbations of symptoms and signs of increased degree of paralysis. The symptoms and signs began to improve and continued uninterruptedly until at the end of two months her muscular impairment of function and mental condition were greatly improved, and she had gained about ten pounds in weight.

May 4 referred to Dr. Brainerd for examination. The following

Fig. 8.—L. W., photograph taken May, 1910, five months after recovery from the active symptoms of measles; eight months after beginning treatment. Note slight strabismus and ptosis of left eye.

is from his record: "May 4, 1911. Patient has been taking tuberculin treatment under Dr. Browning. Weight has gone up to 145 pounds. Mentality and spirits greatly improved, pain in arm better. Condition and use of arm better. Sleeps well. Some recurrence of symptoms after several of the injections of tuberculin.

Patient looks cheerful and bright, talks well. Very little difference in reflexes on the two sides."

She continued under treatment for a year, with steady improvement, until by the end of six months she could come to my office alone. Her muscular system never entirely regained its former condition, but she walks well enough to get around without danger

Fig. 9.—L. W., in good health after 4½ years. Slight external strabismus still exists, the only remaining manifestation of the disease.

or attracting marked attention. Temperature gradually approached normal—97.6-98.4—and her weight increased to 157—gain of 20 pounds. No forced feeding. She gradually lost her sensitiveness to tuberculin and felt much better for about five days following a dose, which was 10 mg. of watery extract. The interval between doses was gradually increased, she being instructed to return whenever she felt toxic. This she did, returning at intervals of from one week to three weeks for another year, until treatment ceased, she having nearly regained her normal condition, and so continues to the present time, about three years later.

This case apparently was a moderately acute meningitis terminating in a chronic form, followed by an arrestment.

Case IV.—September 22, 1909. L. W. Female. Age 2 years 9 months.

Family History.—Youngest of family of three children. Maternal grandmother died as result of accident, other three grandparents living and well—ages 61, 63, 67. Father and mother aged 39 and 37, respectively, two brothers 4 and 7 years. Patient well until "severe cold on lungs," January 18, 1909—two years of age. Coughed very hard, recovery incomplete. July 7, 1909, went on picnic, became exhausted, and grew progressively weaker, appetite poor, cough aggravated, had fever and night sweats.

August 9 mother states left eye disappeared, "went up and out." Consulted oculist. Systemic symptoms grew progressively worse, but eye made some improvement; external strabismus continued. August 13 mother noticed she was losing use of right hand, and that she fell frequently when attempting to walk; soon noticed dragging of left foot, followed by abandoning all efforts to walk. Used electricity for a few days, when noticed "both eyes began to close." August 20 taken to clinic, seen by Drs. Dudley (oculist), Chas. Lewis Allen and Ross Moore (neurologists). Ptosis both eyes, more pronounced in left, with marked degree of paralysis in right arm and hand, slight in left hand, with marked paralysis in lower limbs, was observed. There was evidence of lesion in apices of both lungs, and continuation of symptoms noted above. Syphilis was believed to have been eliminated, and after careful observation of symptoms and signs until September 22 the case was referred to me as probably tubercular meningitis.

Examination showed enlargement of cervical glands, evidence of tubercular involvement of both apices, more marked in right. Moro and Von Pirquet tests promptly and very actively positive. Began the use of watery extract of tubercle bacilli in doses of .000,001 milligram, which was cautiously increased. Several times evidence of focal reaction was observed as noted by temporary exacerbation of physical signs during course of treatment. Marked improvement was manifest at once, as shown by amelioration of symptoms and rapid improvement of signs.

December 19, 1909, the child had an attack of measles, and soon the former signs began to manifest themselves. The signs gradually disappeared, however. Maximum dose of watery extract given was 2 mgs.

May 5, 1911. Continued good health after two years.

The temperature in this case was taken per rectum, and was somewhat erratic. At times it would reach 102, but more frequently was subnormal. The daily range generally did not exceed one and a half degrees.—*Medical Record.*

---

Below we are reprinting an article from the pen of Dr. C. C. Bass, of New Orleans, La.

The prevalence of pyorrhea, the tenacity of the disease, the seeming inability to effect a permanent cure, makes us feel that this article should be placed in the hands of every physician in Texas, and we are reprinting it from the *South Texas Medical Record* in the hope that all independent journals will give it space.

And this leads us to ask, how much longer our leaders and thinkers are going to bury knowledge that should be given to every man in the profession.

Is it not time that the spirit of brotherly kindness and a just pride in a more enlightened profession should cause men to place these helpful articles where all the profession shall profit by them?

MRS. DANIEL.

## Figures and Tables

**Fig. 1. f1:**
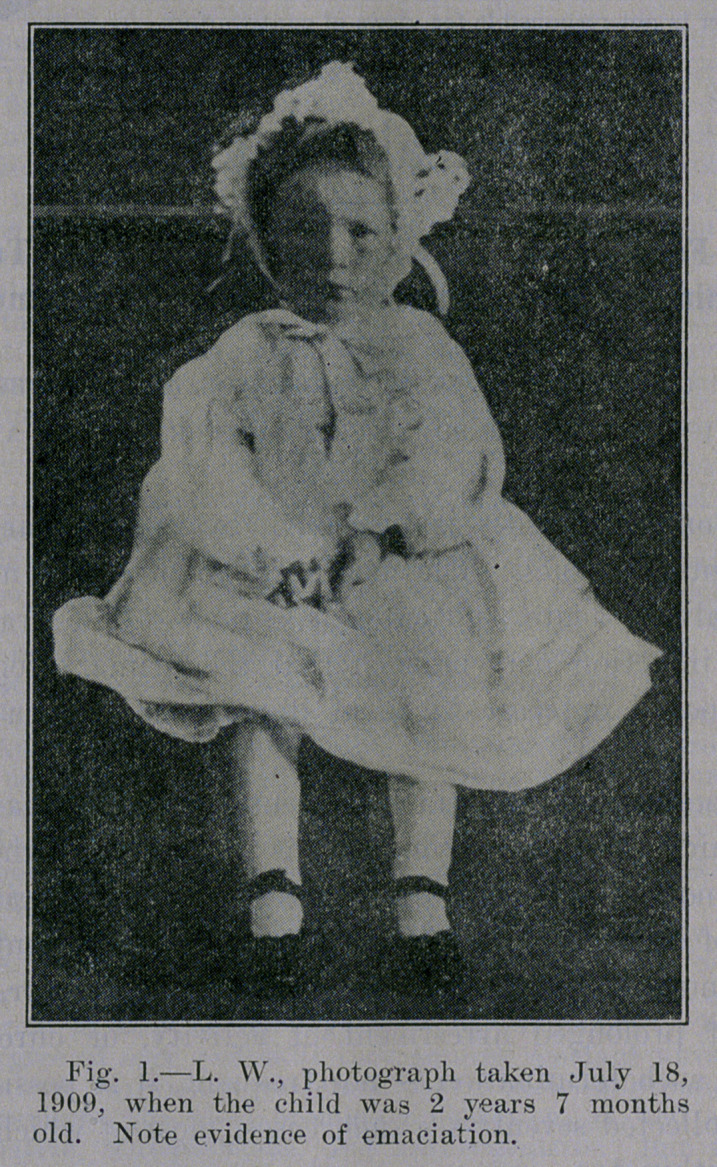


**Fig. 2. f2:**
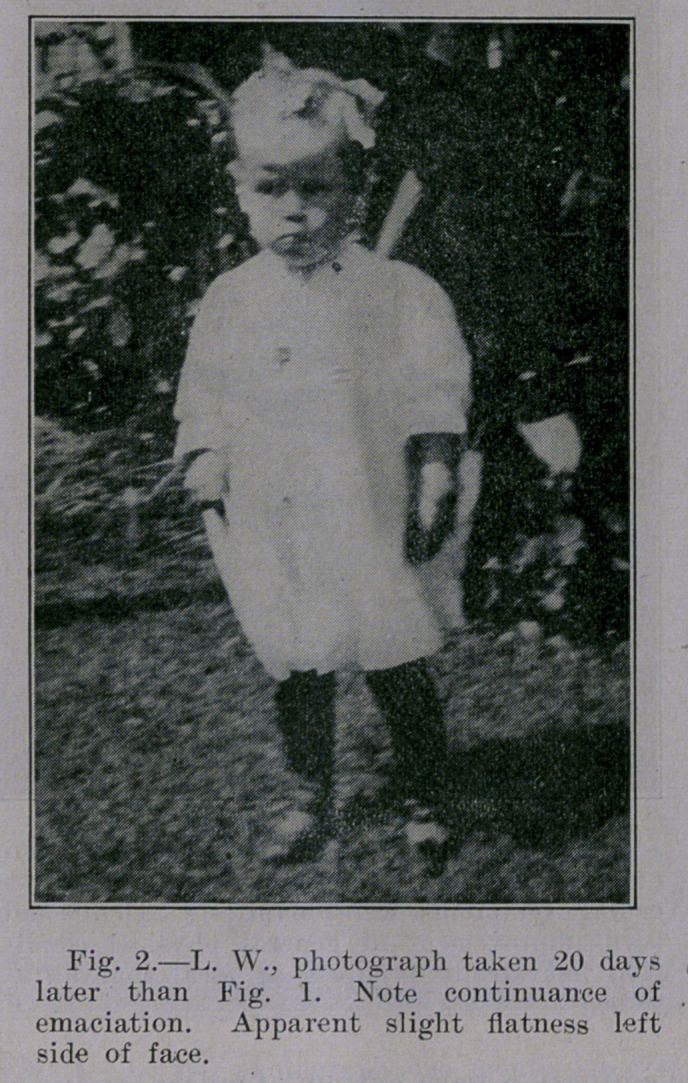


**Fig. 3. f3:**
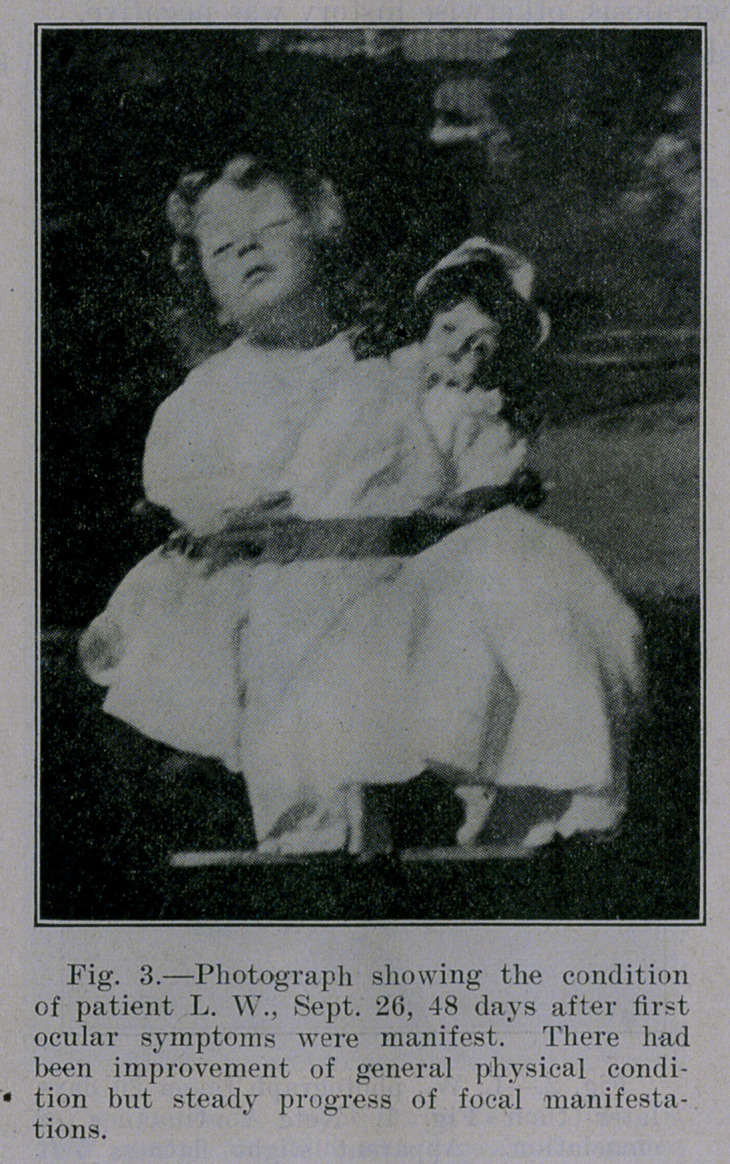


**Fig. 4. f4:**
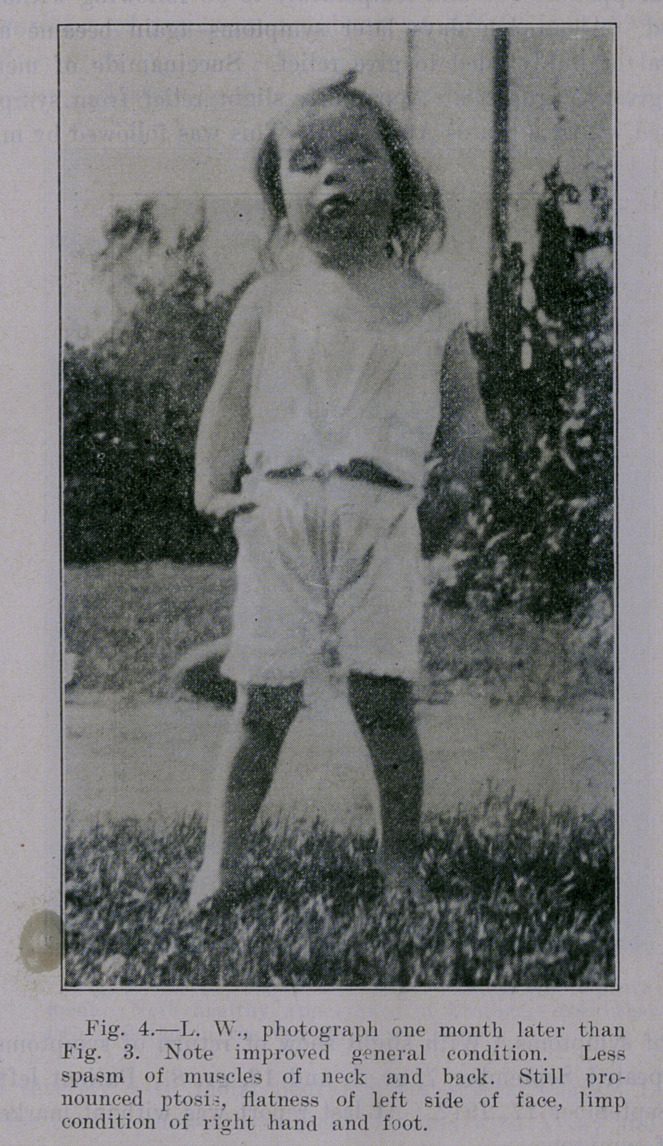


**Fig. 5. f5:**
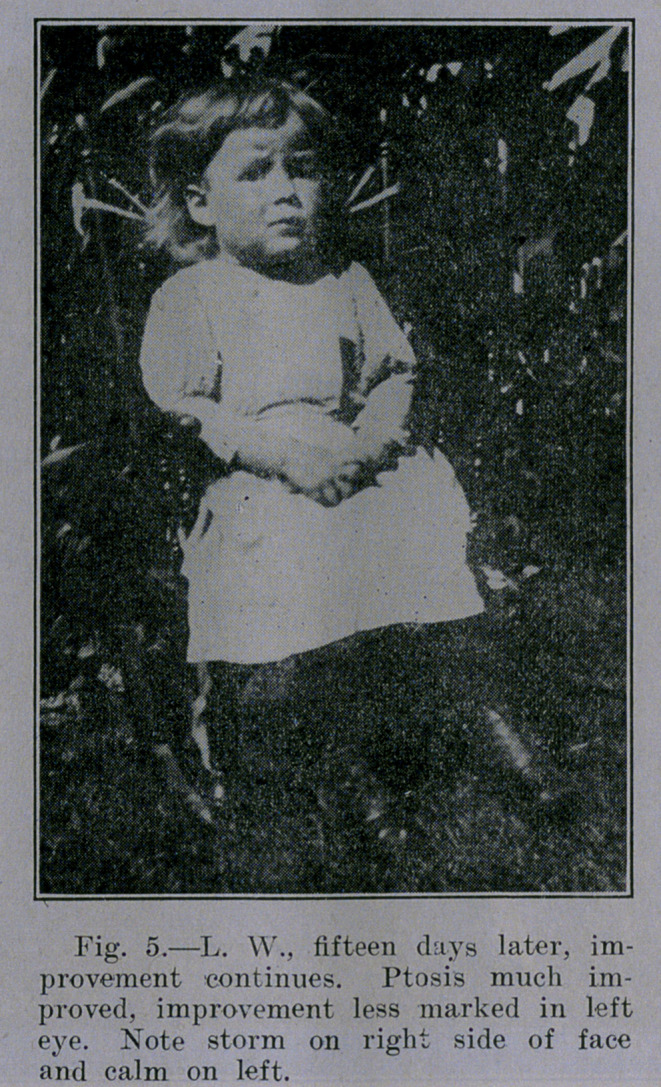


**Fig. 6. f6:**
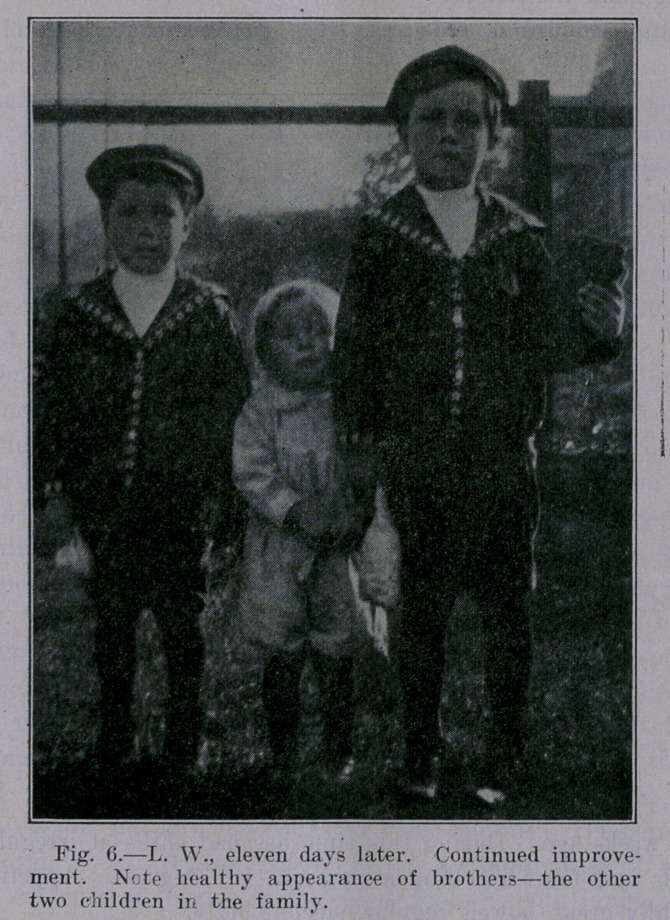


**Fig. 7. f7:**
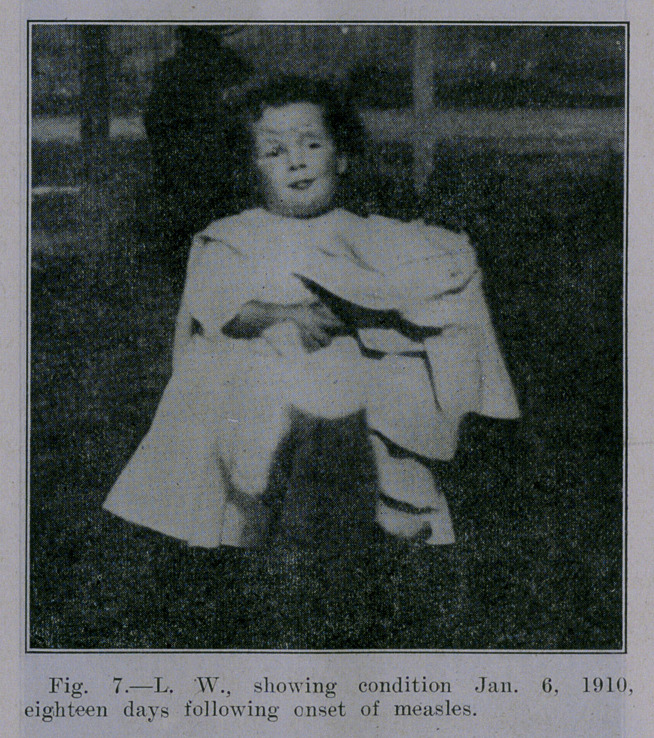


**Fig. 8. f8:**
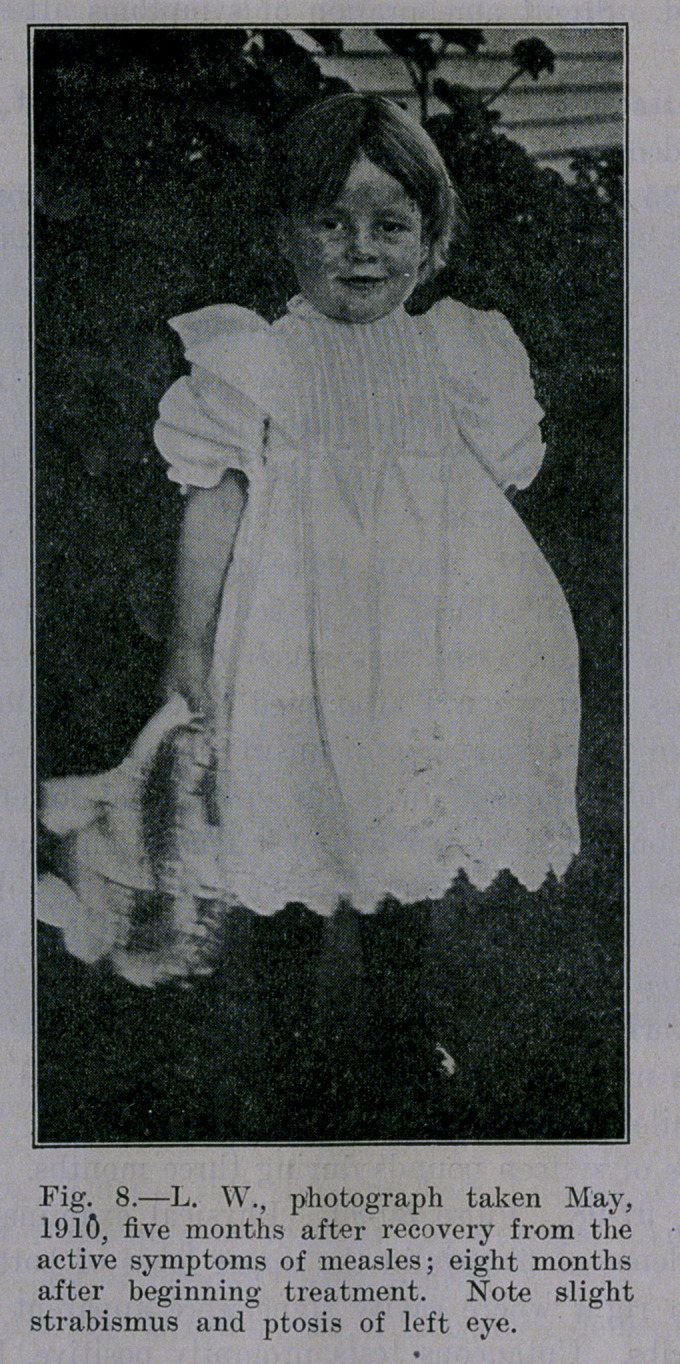


**Fig. 9. f9:**